# Practical Pathology

**Published:** 1887-06

**Authors:** 


*Practical Pathology.* By JOHN LINDSAY STEVEN, M.D. Pp. 266. Glasgow: J. MACLEHOSE & SONS. 1887.

This is a sensible, practical, useful book, dealing with the whole question of the instruction of students in Pathology. Beginning with a full and excellent account of the method of making a *post-mortem* examination, the author gives admirable directions for the preparation, cutting, staining, injecting, and mounting of sections for the microscope, and then gives an exhaustive practical description of morbid histology as regards both processes and organs. The work has evidently been prepared with great care; nothing is superfluous; there is no overlapping of subjects; and the matter has been rigorously subordinated to the wants of the practical student.

It is probably beside the mark to say that we should like to see this work in the hands of every English student who is being instructed in Pathology; for as yet there is scarcely such a thing as pathological instruction in England—at least, in the full sense that such instruction means in Germany and in Scotland. Consequently we fear that the demand for the book on this side of the border will not be on a level with its merits: we trust that its success will be all the more fully assured on its own soil.

